# Primary closure versus vertical rectus abdominis myocutaneous (VRAM) flap closure of perineal wound following abdominoperineal resection—a systematic review and meta-analysis

**DOI:** 10.1007/s11845-024-03651-3

**Published:** 2024-03-27

**Authors:** Hugo C. Temperley, Poorya Shokuhi, Niall J. O’Sullivan, Benjamin Mac Curtain, Caitlin Waters, Alannah Murray, Christina E. Buckley, Maeve O’Neill, Brian Mehigan, Paul H. McCormick, Michael E. Kelly, John O. Larkin

**Affiliations:** 1 Department of Surgery, St. James’s Hospital, Dublin, Ireland; 2 Trinity St James’s Cancer Institute, St. James‘s Hospital, Dublin, Ireland; 3 Department of Surgery, St John of God Subiaco Hospital, Perth, Australia; 4 Department of Surgery, Fiona Stanley Hospital, Perth, Australia; 5 Department of Plastics, St. James’s Hospital, Dublin, Ireland

**Keywords:** Abdominoperineal resection, Perineal complications, Surgical oncology, Vertical rectus abdominis myocutaneous flap

## Abstract

**Purpose/aim:**

Perianal wound healing and/or complications are common following abdominoperineal resection (APR). Although primary closure is commonly undertaken, myocutaneous flap closure such as vertical rectus abdominis myocutaneous flap (VRAM) is thought to improve wound healing process and outcome. A comprehensive meta-analysis was performed to compare outcomes of primary closure versus VRAM flap closure of perineal wound following APR.

**Methods:**

PubMed, MEDLINE, EMBASE, and Cochrane Central Registry of Controlled Trials were comprehensively searched until the 8th of August 2023. Included studies underwent meta-analysis to compare outcomes of primary closure versus VRAM flap closure of perineal wound following APR. The primary outcome of interest was perineal wound complications, and the secondary outcomes were abdominal wound complications, dehiscence, wound healing time, length of hospital stay, and mortality.

**Results:**

Ten studies with 1141 patients were included. Overall, 853 patients underwent primary closure (74.8%) and 288 patients underwent VRAM (25.2%). Eight studies reported on perineal wound complications after APR: 38.2% (*n* = 263/688) in the primary closure group versus 32.8% (*n* = 80/244) in the VRAM group. Perineal complication rates were statistically significantly lower in the VRAM group versus primary closure ((M-H OR, 1.61; 95% CI 1.04–2.49; <*p* = 0.03).

**Conclusion:**

We highlight the advantage of VRAM flap closure over primary closure for perineal wounds following APR. However, tailoring operative strategy based on patient and disease factors remains important in optimising outcomes.

**Supplementary Information:**

The online version contains supplementary material available at 10.1007/s11845-024-03651-3.

## Introduction

Abdominoperineal resection (APR) remains a common, definitive surgical component of the primary treatment of anorectal cancer [1]. Historically, perineal and midline abdominal incisions are utilised to excise the distal colon, rectum, and anal sphincter complex, with fashioning an end colostomy. The complexity of this operation and potential for surgical complications in the post-operative period are often underestimated [1]. Intuitively, complications can occur in either the abdominal or perineal spaces. Intraabdominal abscess, post-operative ileus, and mechanical obstruction are two well-recognised intra-abdominal sequalae of APR, due to empty pelvis syndrome [2]. Perineal complications are more prevalent, including poor wound healing (86%), infection (27%), wound dehiscence (22%), and/or pelvic collections (7%) [3]. Minimally invasive (laparoscopic/robotic) APR techniques are increasingly performed, with excellent outcomes [4, 5].

Although primary closure (PC) has been common practise, the associated rate of perineal wound complications ranges from 25 to 60% [6]. One of the main contributors to perineal wound complications is the large dead space (empty pelvis syndrome) in the pelvic cavity after an oncological resection [7]. Factors such as neoadjuvant therapy, smoking, and diabetes also negatively impact the wound-healing process [8].

In order to mitigate the risk of incurring these perineal complications, alternative closure methods are required, as opposed to PC, especially as the majority of patients will have received chemoradiotherapy [9]. Abdominal-based flaps such as VRAM, transverse rectus abdominis (TRAM), or deep inferior epigastric artery perforator (DIEP) have been utilised. These flaps usually involve a non-irradiated blood supply, however, may increase operative times [10]. Alternatively, thigh-based flaps (gracilis, anterolateral, and gluteal) are also a good option, especially for smaller wounds.

Myocutaneous flap reconstruction of the perineum, such as the vertical rectus abdominis myocutaneous (VRAM) flap, has been shown to be a valuable alternative to primary closure [11]. The theory is that flap reconstruction not only fills the pelvic dead space but it also thought to improve wound healing and reduce wound infection as a function of its good vascular supply [11, 12].

VRAM involves making an elliptical incision from the inframammary fold at the midline to around the level of the umbilicus. This flap is then harvested with the rectus abdominus and anterior rectus sheath above the arcuate line and with the rectus abdominus alone below it. The flap is then transposed and rotated no more than 180 degrees, into the perineal wound [6]. When compared to PC, VRAM has been observed to improve wound healing and lower the risk of developing a pelvic abscess [13]. Overall, complication rates of 17.8% have been described, with 3.5% of flap patient requiring re-intervention [6]. This review will comprehensively synthesise the available literature in relation to post-operative outcomes, comparing VRAM with primary closure.

## Methods

### Registration and search strategy

**Fig. 1:**
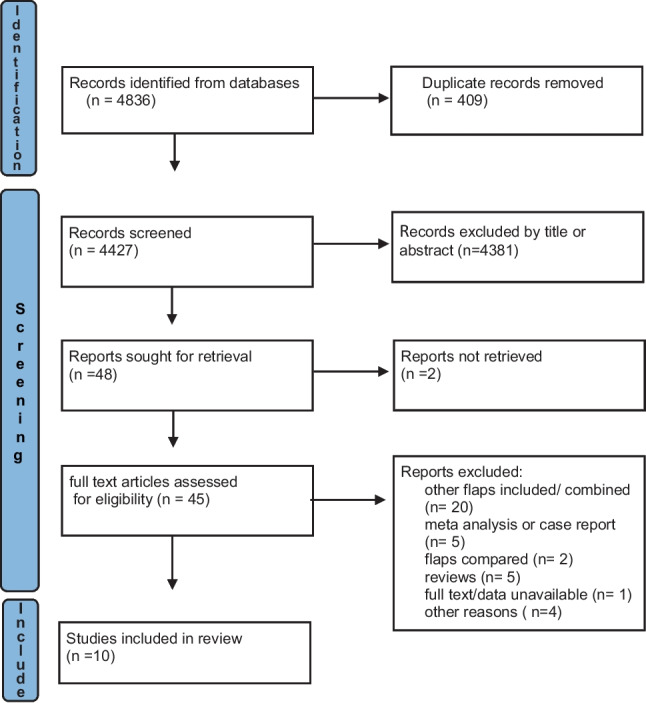
PRIMSA flowchart outlining the systematic search process

Our search was conducted in line with the most recent PRISMA recommendations [14]. Our study protocol was prospectively registered with PROSPERO under the following registration number: CRD42023456092. We conducted a search using PubMed, MEDLINE (Ovid), EMBASE, and Cochrane Central Register of Controlled Trials using a search strategy undertaken on August 8, 2023. The search pathway has been illustrated in the PRISMA diagram Fig. 1. The grey literature was also searched for any relevant studies. The systematic search process with detailed search terms is outlined in Supplementary material 1 (S1).

### Inclusion/exclusion criteria

#### Inclusion criteria

In order to be included in our analysis, the studies had to meet the following criteria: (a) report on patients with colorectal cancer who underwent APR, (b) be a comparative study in regards to PC vs VRAM flap, (c) report on surgical outcomes and complications, and (d) have a well-defined research methodology.

#### Exclusion criteria

Studies were excluded from the analysis if: (a) they did not separately analyse the type of the flap used for reconstruction, (b) they were not comparative studies, (c) they did not have matched patient populations, (d) the outcomes of interest were not reported, and (e) the methodology was not clearly reported.

### Identification of studies and outcomes of interest

Studies that satisfied the inclusion and exclusion criteria were included in our review. The following PICO elements were used as the basis for selecting studies.

- Population: patients undergoing abdominoperineal resection for colorectal/anal cancer
- Intervention: VRAM
- Comparison: primary closure
- Outcome:
  - Primary outcome: perineal wound complications after APR
  - Secondary outcomes:
    -  Perineal wound complications
    -  Dehiscence of wound
    -  Wound healing time (days)
    -  Length of hospital stay (days)
    -  Mortality within 30 days

### Study selection, data extraction, and critical appraisal

A database was created using the reference managing software EndNote X9 TM. Abstracts of articles yielded from the search were reviewed by two independent authors (HCT and NOS) based on the inclusion and exclusion criteria detailed above. Following the removal of duplicate articles, discrepancies in judgement about the relevance of articles were resolved via an open discussion between the authors and an independent third reviewer (MK). An article was excluded from the review when the three reviewers came to an agreement. Full-texts of short-listed articles were obtained and further evaluated to ensure that they met our inclusion criteria. The references of short-listed articles were then searched to identify other relevant studies that may have been missed through the initial search of online databases. Data were extracted by two reviewers independently from the articles that met inclusion criteria based on full-text review. Information extracted was based on the PICOTS framework (Population, Intervention, Comparator, Outcomes, Timing, and Setting) [15]. In order to extract and store data efficiently, the Cochrane Collaboration screening and data extraction tool, Covidence, was used [16]. Conflicts between the two reviewers were resolved following an open discussion and final decision by the senior author (MK).

### Risk of bias

Assessment of potential biases within included RCTs was assessed using the Cochrane Collaboration (for randomised controlled trials [RCTs]) [17]. This assessment tool grades each study as being high (red circle), low (green circle), or unclear (yellow) risk of bias across six categories. For non-RCT studies, Newcastle–Ottawa scale (HT) risk of bias tool was used and the results tabulated [18]. This assessment tool grades each study as being ‘satisfactory’ or ‘unsatisfactory’ across various categories. We assigned stars to evaluate study quality: 7 stars ‘very good’, 5–6 stars ‘good’, 3–4 stars ‘satisfactory’, and 0–2 stars ‘unsatisfactory’. The critical appraisal was completed by two reviewers independently (HT and NOS), where once again, a third reviewer (MK) was asked to arbitrate in cases of discrepancies in opinion.

### Statistical analysis

Statistical analysis was performed using Revman Statistical Software (Ver. 5 Copenhagen, Denmark). Binary outcome data were reported as odd ratios (OR), and 95% confidence interval (95% CI) was estimated using the Mantel–Haenszel method. For continuous data, mean differences and 95% CI were estimated using inverse variance weighting. Outcome measures (mean + standard deviation and median + inter-quartile range) were recorded. If needed, outcome variables (mean and SD) were estimated from the median and range using formula described by Hozo et al. [19]. Statistical significance was attributed to *p* value <0.05.

Perineal wound, abdominal wound complications, dehiscence, and mortality were expressed as dichotomous or binary outcomes, reported as OR, expressed with 95% CI. ORs were calculated, using crude event data, to compare interventions using per-protocol data, where applicable. Wound healing time and length of hospital stay were calculated using mean values, SD, and pooled mean variance.

## Results

### Search results

In total, 4836 articles were identified and 409 duplicate articles were excluded. Thereafter, study titles and abstracts were screened, resulting in 45 studies being eligible for full-text review. Of these, ten studies met the eligibility criteria and were included [13, 20–28]. The PRISMA flowchart is illustrated in Fig. 1. Overall, 9/10 studies are comparative retrospective studies with the remaining study being a randomised control trial (RCT) [27].

### Study characteristics

Overall, 1141 patients undergoing APR were included, of whom 853 patients underwent PC (74.9%) and 288 patients underwent VRAM (25.2%). The mean (range) age at surgery was 58.9 years (53.5–66.7 years). In total, 59.8% (*n* = 682/1141) were male patients and 40.2% (*n* = 459/1141) were female. The oncological lesion location was low rectal only in 4/10 studies [20, 25, 13, 27], rectal and anal in 5/10 [21, 22, 24, 26, 28], and anal only in 1/10 [23]. The characteristics of the trials included in this meta-analysis are shown in Table 1.

**Table 1:** Characteristics of the trials included in this meta-analysis

**Author**	**Country**	**Study design**	**Closure (*n*)**		**Age**		**Gender M:F**		**Lesion site**
			**Primary**	**VRAM**	**Primary**	**VRAM**	**Primary**	**VRAM**	
Althumairi et al. [20]	USA	Comparative, retrospective	56	11	57	57	35:21	4:7	Rectal
Butler et al. [21]	USA	Comparative, retrospective	76	35	56.2	54.3	53:23	7:28	Rectal + anal
Chessin et al. [22]	USA	Comparative, retrospective	59	19	60.3	56.5	38:21	2:17	Rectal + anal
Lefevre et al. [23]	France	Comparative, retrospective	52	43	62.1	56.3	13:39	12:31	Anal
Nichols et al. [24]	USA	Comparative, retrospective	129	29	61.0	58.7	89:40	15:14	Rectal + anal
O’Dowd et al. [25]	Ireland	Comparative, retrospective	27	12	61.5	66.7	22:5	7:5	Rectal
Spasojevic et al. [13]	Norway	Comparative, retrospective	260	69	64	64	186:74	19:50	Rectal
Touny et al. [27]	Egypt	Prospective randomized controlled study	30	30	51	53.5	24:6	21:9	Rectal
Woodfield et al. [28]	New Zealand	Comparative, retrospective	37	31	56	63	21:16	10:21	Rectal + anal
Sheckter et al. [26]	USA	Comparative, retrospective	127	51	61.04	57.94	81:46	22:29	Rectal + anal

Overall, 7/10 studies examined the stage of cancer [13, 20–23, 25, 27, 29]. In total, 50.1% (271/530) of patients in the primary group were stage I–II, with the remainder being stage III–IV (43.9%) 230/530). In comparison, in the VRAM group, 46.9% (121/258) were stage I–II, with the remained being stage III–IV ((53.1%) 137/258). Six studies examined neoadjuvant chemotherapy, with 65.0% (228/351) in the PC group versus 69.3% (115/166) in the VRAM group [20, 22, 23, 25–27, 30]. Eight studies examined neoadjuvant radiotherapy, with 75.4% (562/745) in the PC group versus 89.3% (225/252) in the VRAM group [13, 20, 22, 25–28]. Five studies examined smoking status, with 31.9% (194/609) in the PC group versus 22.5% (43/191) in the VRAM group [20, 24, 26, 13, 28]. Five studies reported on diabetes status, with 14.7% (65/442) in the PC group versus 16.3% (26/160) in the VRAM group [20, 13, 22, 27, 28]. The patient characteristics of the trials included in this meta-analysis are shown in Table 2.

**Table 2:** The patient characteristics of the trials included in this meta-analysis

Author	**Stage I–II**		**Stage III–IV**		**Neoadjuvant chemo**		**Neoadjuvant radiation**		**Smoking**		**DM**	
	**1’**	**VRAM**	**1’**	**VRAM**	**1’**	**VRAM**	**1’**	**Flap**	**1’**	**Flap**	**1’**	**Flap**
Althumairi et al. [20]	20	4	36	7	45	10	43	10	22	3	5	0
Butler et al. [21]	15	46	10	29	/	/	/	/	/	/	/	/
Chessin et al. [22]	43	9	16	10	59	19	59	19	/	/	10	0
Lefevre et al. [23]	32	14	20	29	35	31	/	/	/	/	/	/
Nichols et al. [24]	/	/	/	/	/	/	61	18	29	13	/	/
O’Dowd et al. [25]	12	4	15	8	11	10	20	12	/	/	/	/
Spasojevic et al. [13]	134	27	118	41	/	/	260	69	83	17	36	11
Touny et al. [27]	15	17	15	13	0	0	30	30	/	/	8	11
Woodfield et al. [28]	/	/	/	/	/	/	11	22	17	10	6	4
Sheckter et al. [26]	/	/	/	/	78	45	78	45	43	/	/	/

### Primary and secondary outcomes (Table 3)

**Table 3:** Outcome measures examined in this meta-analysis

**Author**	**Wound healing time (days) (mean + SD)**		**Abdominal complications**		**Perineal complications**		**Dehiscence**		**LOS (days) (mean + SD)**		**Mortality**	
	**1’**	Flap	**1’**	Flap	**1’**	Flap	**1’**	Flap	**1’**	Flap	**1’**	Flap
Althumairi et al. [20]	47.6 (46.9)	44.1 (31.5)	11/56	1/11	21/56	3/11	/	/	9.3 (5)	13.4 (9)	3/56	1/11
Butler et al. [21]	/	/	25/76	10/35	35/76	16/35	32/76	12/35	/	/	0/0	0/0
Chessin et al. [22] 5919	39.5 (255)	81.5 (52.5)	9/59	2/19	26/59	3/19	/	/	/	/	/	/
Lefevre et al. [23]	95.1 (113.2)	18.7 (34.9)			19/43	11/41	/	/	23.1 (17.2)	25.1 (10.7)	0/52	2/43
Nichols et al. [24]	/	/	/	/	/	/	13/129	7/29	/	/	/	/
O’Dowd et al. [25]	/	/	/	/	/	/	9/27	3/12	19.5 (3.1)	19.0 (13.2)	/	/
Spasojevic et al. [13]	/	/	/	/	94/260	25/69	94/260	25/69	12 (11)	19 (16.75)	3/260	1/69
Touny et al. [27]	24 (5.75)	25.2 (2.3)	5/28	6/28	14/30	5/29	8/30	2/20	9 (10.75)	7 (9.25)	2/30	2/30
Woodfield et al. [28]	58.8 (59.1)	58.2 (17.1)	/	/	21/37	17/31	/	/	13 (8.50)	13 (12)	/	/
Sheckter et al. [26]	/	/	8/127	0/9	33/127	0/9	9/127	0/9	/	/	/	/

#### I. Perineal wound complications

**Fig. 2:**
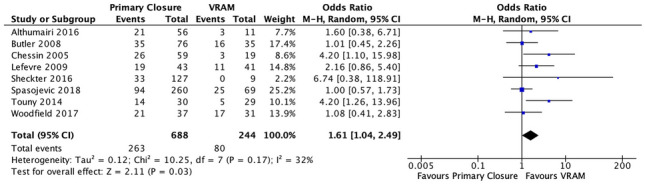
Perineal wound complications meta-analysis results

Eight studies reported on perineal wound complications after APR; 38.2% (263/688) in the PC group versus 32.8% (80/244) in the VRAM group [20–22, 13, 23, 26–28, 31]. A meta-analysis using the M-H random-effects model revealed a significant difference in perineal complication rates between the two groups, with a significantly lower rate in the VRAM group (M-H OR, 1.61; 95% CI 1.04–2.49; < *p* = 0.03) (Fig. 2).

#### II. Abdominal wound complications

**Fig. 3:**
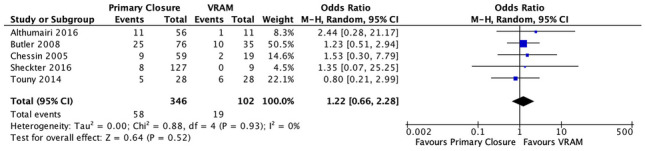
Abdominal wound complications meta-analysis results

Five studies reported on abdominal wound complications after APR; 16.8% (58/346) in the PC group versus 18.6% (19/102) in the VRAM group [20–22, 26, 27]. A meta-analysis using the M-H random-effects model revealed no significant difference between the two groups (M-H OR, 1.22; 95% CI 0.66–2.28; *p* = 0.52) (Fig. 3).

#### III. Dehiscence

**Fig. 4:**
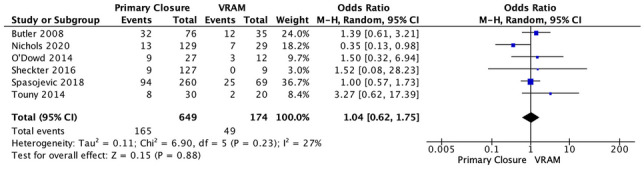
Dehiscence meta-analysis results

Six studies reported on perineal wound dehiscence after APR; 25.4% (165/649) in the PC group versus 28.2% (49/174) in the VRAM group [13, 21, 24–27]. A meta-analysis using the M-H random-effects model revealed no significant difference rates between the two groups (M-H OR, 1.04; 95% CI 0.62–1.75; *p* = 0.88) (Fig. 4).

#### IV. Wound healing time (days)

**Fig. 5:**
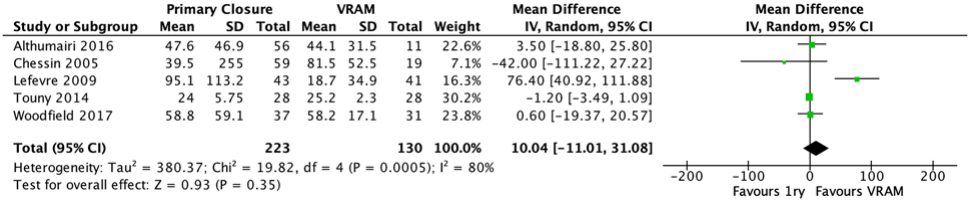
Wound healing time (days) meta-analysis results

Five studies reported on wound healing time [20, 22, 23, 27, 28]. Overall, VRAM had a shorter healing time (weighted mean 40.2 days, SD 37.7), compared with PC (weighted mean 54.9 days, SD 143.6). A meta-analysis performed using the random-effects model revealed no significant difference in wound healing time between the two groups (MD 10.04 95% CI −11.01–31.08; *p* = 0.35) (Figs. 5, S2 and S3).

#### V. Length of stay (days)

**Fig. 6:**
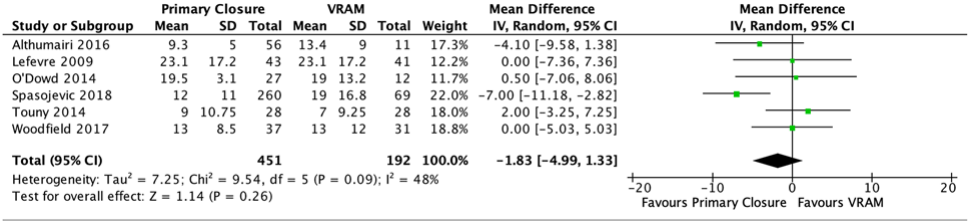
Length of stay (days) meta-analysis results

Five studies reported on postoperative length of stay [13, 20, 23, 25, 27, 28]. Overall, PC had a shorter LOS weighted mean (12.7 days, SD 11.8), compared with VRAM (weighted mean 17.2 days, SD 14.3). A meta-analysis performed using the random-effects model revealed no significant difference in length of stay between the two groups (MD − 1.83 95% CI −4.99–1.33; *p* = 0.26) (Figs. 6, S4 and S5).

#### VI. Mortality

**Fig. 7:**
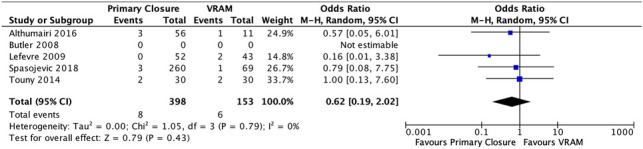
Mortality meta-analysis results

Four studies reported on mortality after APR; 2.0% (8/398) in the PC group versus 3.92% (6/153) in the VRAM group [20, 21, 23, 13, 27]. A meta-analysis using the M-H random-effects model revealed no significant difference rates between the two groups (M-H OR, 0.62; 95% CI 0.19–2.02; *p* = 0.43) (Fig. 7).

### Risk of bias

The only included RCT was ‘low risk’ of bias for most of the categories, using the Cochrane Collaboration risk of bias assessment for RCTs. In regards to non-RCT studies, two studies were seven ‘very good’, zero studies were ‘good’, zero studies were ‘satisfactory’, and zero studies were ‘unsatisfactory’. S6/7 summarises the results of our risk of bias assessment and individual breakdown of included studies.

## Discussion

The management of perineal wounds following APR has long been a subject of concern amongst surgeons due to the potential for complications and delayed wound healing [7, 30, 31]. The choice between PC and myocutaneous flap closure, such as the VRAM, has been a matter of debate [29]. Our findings suggest that myocutaneous flap closure with VRAM appears to lead to better perineal wound outcomes, without a rise in abdominal wall complications. These findings suggest that perineal closure techniques should be discussed in advance of surgical intervention at multidisciplinary team meetings for individual patients.

Achieving clear resection margins in APR for anorectal malignancies is challenging [32]. While the goal is to remove cancerous tissue effectively, this often results in creating sizeable pelvic dead space [33]. This area forms non-collapsible, dead space within the pelvis, which is susceptible to clinical complications such as fluid buildup, infections, and compromised healing of perineal wounds (empty pelvis syndrome) [34, 35]. The risk of these complications is greater in cases where patients have undergone neoadjuvant radiotherapy, further contributing to difficulties in the perineal region [36–38]. In our review, 75.4% in the PC group and 89.3% (225/252) in the VRAM group had received neoadjuvant radiotherapy. One study found an upwards of 40% increase in perineal wound complications when patients undergoing APR had previously had radiation [39].

We acknowledge that our review does have some minor limitations. A single RCT was included in this review, with the remainder being retrospective comparative studies [27]. Consequently, the level of evidence presented is not based solely on high-quality randomised trials; however, the included comparative articles serve as the best available evidence at this time. Additionally, the limitations of meta-analyses in general should be taken into account [40]. Despite this, our study provides important data for the shared decision-making process. Future studies should also focus on quality of life outcomes in patients undergoing PC or VRAM post-APR. Nevertheless, RCTs comparing PC to VRAM may be not ethically suitable or clinically required to demonstrate the benefits of this technique. Notwithstanding, this study will impact clinical practise by allowing surgeons to counsel patients appropriately on the optimal managements options and inform patient on expected outcomes.

## Conclusion

This review highlights the benefit that VRAM flap closure offers over PC in terms of perineal wound complications. Despite these results, clinical decision-making should remain patient-centred, taking into account patient and disease factors.

### Supplementary Information

Below is the link to the electronic supplementary material.

Supplementary file1 (DOCX 373 KB)
