# Supplementary material for: Primary closure versus vertical rectus abdominis myocutaneous (VRAM) flap closure of perineal wound following abdominoperineal resection—a systematic review and meta-analysis
Source: Ir J Med Sci. 2024 Mar 27;193(4):1721–8. doi: 10.1007/s11845-024-03651-3 (PMC11294374; doi:10.1007/s11845-024-03651-3)
Supplement: Supplementary file 1 — Supplementary file1 (DOCX 373 KB) [file 11845_2024_3651_MOESM1_ESM.docx]

Combinations of the following Medical Subject Headings (MeSH) and non-MeSH terms were used: ‘rectal’, ‘rectum’, ‘anal’, ‘anal canal’, ‘anus’, ‘colorectal’; ‘cancer’, ‘carcinoma’, ‘tumour’, ‘tumor’, ‘neoplasms’, ‘squamous cell carcinoma’; ‘abdominoperineal resection’, ‘abdominoperineal excision’, ‘perineum or perineal surgery’, ‘perineum or perineal wound’, ‘APR’; ‘perineum or perineal wound healing’, ‘perineum or perineal wound repair’; ‘flaps’, ‘myocutaneous flap’, ‘myocutaneous flaps reconstruction’, ‘flaps reconstruction’, ‘primary closure’, ‘primary suture’.

**Supplementary material 1 (S1)**

**
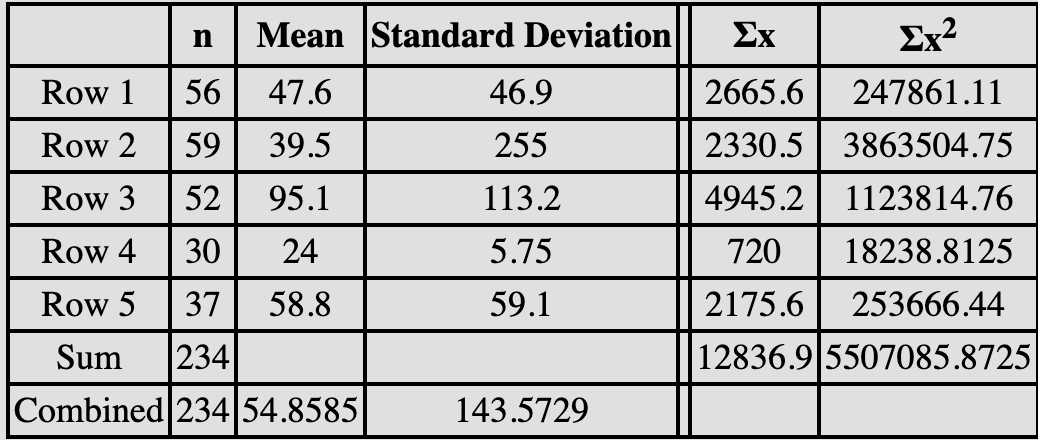
**

**Supplementary material 2 (S2): Wound healing time (days) (primary closure) - Combined groups of means and Standard Deviations into a Single Group by Decomposing Mean and SD – for continuous data**

**
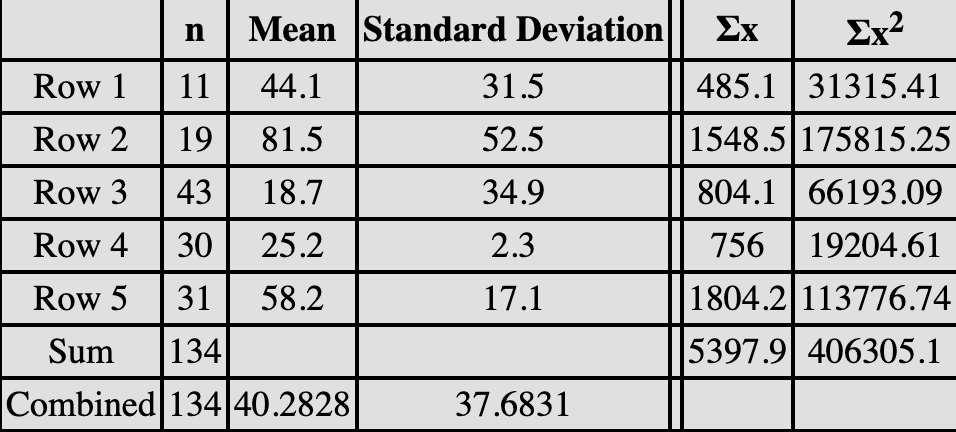
**

**Supplementary material 3 (S3): Wound healing time (days) (VRAM) - Combined groups of means and Standard Deviations into a Single Group by Decomposing Mean and SD – for continuous data**

**
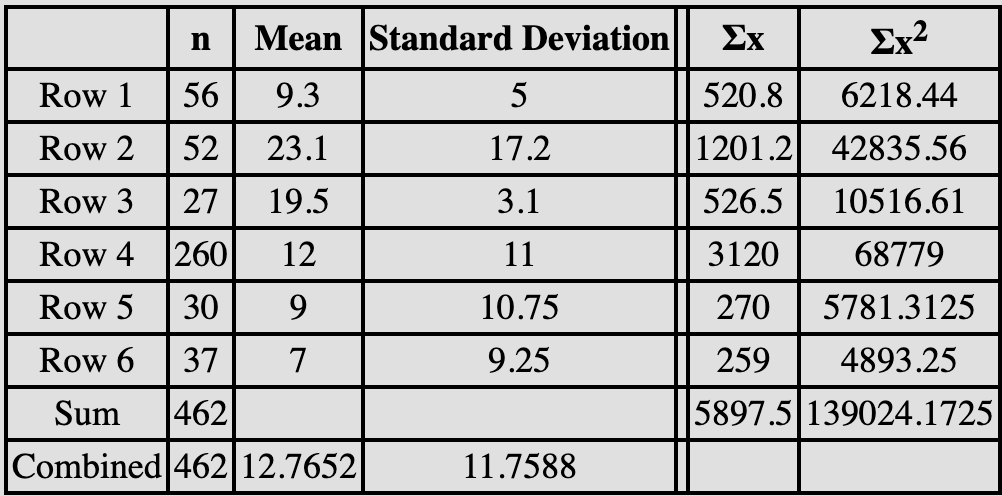
**

**Supplementary material 4 (S4): Length of stay (Primary closure) - Combined groups of means and Standard Deviations into a Single Group by Decomposing Mean and SD – for continuous data**

**
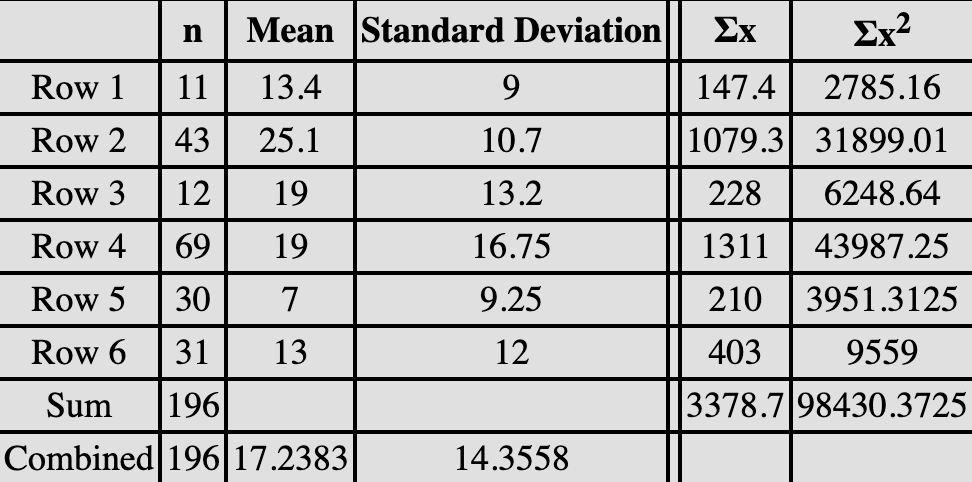
**

**Supplementary material 5 (S5): Length of stay (VRAM) - Combined groups of means and Standard Deviations into a Single Group by Decomposing Mean and SD – for continuous data**

**Supplementary appendix 6: Risk of bias assessment (Cochrane collaboration risk of bias tool for RCTs)**

| **Author** | **Random sequence generation (SB)** | **Allocation concealment (SB)** | **Blinding of participants and**  **personnel (PB)** | **Blinding of outcome**  **assessment (DB)** | **Incomplete outcome data (AB)** | **Selective reporting (RB)** | **Other bias** |
| --- | --- | --- | --- | --- | --- | --- | --- |
| Touny, 2014 | unclear | + | unclear | unclear | + | + | + |

| Author | **Selection** | | | | **Comparability** | **Outcome** | | | **Quality** |
| --- | --- | --- | --- | --- | --- | --- | --- | --- | --- |
|  | **Representativeness of the exposed cohort** | **Sample size (<25 = no star)** | **Non-respondents** | **Ascertainment of the exposure** | **The subjects in different outcome groups are comparable** | **Assessment of outcome** | **Statistical test** | **Period (<4weeks)** |  |
| Althumairi, 2016 | **🟑** | **🟑 ** | / | **🟑** | **🟑** | / | **🟑** | / | 5 |
| Butler, 2008 | **🟑** | **🟑 ** | / | **🟑** | **🟑** | **🟑** | **🟑** | **🟑** | 7 |
| Chessin, 2005 | **🟑** | **🟑** | / | / | **🟑** | **🟑** | / | **🟑** | 5 |
| Lefevre, 2009 | **🟑** | **🟑 ** | / | / | **🟑** | **🟑** | / | **🟑** | 5 |
| Nichols, 2020 | **🟑** | **🟑** | **🟑** | / | **🟑** | / | **🟑** | **🟑** | 6 |
| O’Dowd, 2014 | **🟑** | **🟑** | **🟑** | / | **🟑** | / | **🟑** | **🟑** | 6 |
| Spasojevic, 2018 | **🟑** | **🟑** | **🟑** | / | **🟑** | / | **🟑** | / | 5 |
| Touny, 2014 | **🟑** | **🟑** | / | / | **🟑** | /**🟑** | **🟑** | / | 5 |
| Woodfield, 2017 | **🟑** | **🟑** | / | / | / | **🟑** | **🟑** | **🟑** | 6 |
| Sheckter, 2016 | **🟑** | **🟑** | **🟑** | **🟑** | **🟑** | **🟑** | **🟑** | **🟑** | 7 |

**Supplementary appendix 7: risk of bias assessment (Newcastle-Ottawa scale)**
